# Pathological Changes in Hepatic Sinusoidal Endothelial Cells in *Schistosoma japonicum*-Infected Mice

**DOI:** 10.3390/tropicalmed8020124

**Published:** 2023-02-17

**Authors:** Tingting Jiang, Xiaoying Wu, Hao Zhou, Yuan Hu, Jianping Cao

**Affiliations:** 1 National Institute of Parasitic Diseases, Chinese Center for Disease Control and Prevention, (Chinese Center for Tropical Diseases Research), Key Laboratory of Parasite and Vector Biology, National Health Commission of People’s Republic of China, World Health Organization Collaborating Center for Tropical Diseases, Shanghai 200025, China; 2 The School of Global Health, Chinese Center for Tropical Diseases Research, Shanghai Jiao Tong University School of Medicine, Shanghai 200025, China

**Keywords:** *Schistosoma japonicum*, liver sinusoidal endothelial cells, de-differentiation, epithelial–mesenchymal transition, liver fibrosis

## Abstract

Schistosomiasis japonica is a zoonotic parasitic disease causing liver fibrosis. Liver sinusoidal endothelial cells (LSECs) exhibit fenestrations, which promote hepatocyte regeneration and reverses the process of liver fibrosis. To investigate the pathological changes of LSECs in schistosomiasis, we established a Schistosomiasis model. The population, phenotype, and secretory function of LSECs were detected by flow cytometry at 20, 28, and 42 days post infection. The changes in LSEC fenestration and basement membrane were observed through scanning electron microscopy (SEM) and transmission electron microscopy (TEM). Quantitative real-time PCR and Western blotting were used to detect the expression of molecules associated with epithelial–mesenchymal transition (EMT) and fibrosis of LSECs and the liver. The flow cytometry results showed that the total LSEC proportions, differentiated LSEC proportions, and nitric oxide (NO) secretion of LSECs were decreased, and the proportion of dedifferentiated LSECs increased significantly post infection. The electron microscopy results showed that the number of fenestrate was decreased and there was complete basement membrane formation in LSECs following infection. The qPCR and Western blot results showed that EMT, and fibrosis-related indicators of LSECs and the liver changed significantly during the early stages of infection and were aggravated in the middle and late stages. The pathological changes in LSECs may promote EMT and liver fibrosis induced by *Schistosoma japonicum* infection.

## 1. Introduction

Schistosomiasis japonica is a zoonotic parasitic disease. It is primarily prevalent in China, the Philippines, and other Asian countries. Eggs are deposited in the host liver, causing portal hypertension, liver fibrosis, and splenomegaly, seriously threatening human health [1]. By the end of 2020, there were still 450 endemic counties (cities and districts) in China, 29,517 cases of advanced schistosomiasis, and approximately 71,370,400 people at risk of infection [2]. After several efforts, schistosomiasis infection has been well controlled in China. Infection control was achieved in 2008, and transmission control was completed in 2015 [3]. In contrast, the risk of transmission remains high due to several hosts and the ecological environment for snail breeding [4]. Schistosomiasis remains endemic in some areas of China [5]. Moreover, there are still many chronic schistosomiasis patients in China [2]. In February 2022, the WHO published new guidelines to update the global public health strategy against schistosomiasis [6]. Due to a lack of effective treatment for schistosomiasis liver fibrosis, there is an urgent need to study the regulatory mechanism of liver fibrosis.

Chronic liver injury leads to liver inflammation and fibrosis, which activates myofibroblasts and leads to the secretion of extracellular matrix proteins [7]. Liver sinusoidal endothelial cells (LSECs) in the hepatic sinuses are the first cells to respond to liver injury [8,9]. As a barrier between hepatocytes and blood flow, LSECs have fenestrations due to a lack of a basal membrane, which can promote nutrient transport [8]. Under physiological conditions, LSECs can regulate hepatic vascular tension and help maintain low portal pressure [9]. LSECs maintain hepatic stellate cells and Kupffer cells in the resting state and promote immune tolerance in the liver [8]. Previous research has shown that LSECs are involved in initiating hepatocyte regeneration and reversing the process of liver fibrosis. In contrast, abnormal activation of LSECs associated with chronic liver injury can also induce liver fibrosis [10].

There are two phenotypes of LSECs, including differentiated and dedifferentiated phenotypes. Differentiated LSECs have abundant fenestral structures and lack a complete basement membrane, which is essential to inducing hepatocyte regeneration and maintaining HSCs in a resting state [9]. Dedifferentiated LSECs lose their fenestral structure and form a complete basement membrane beneath the cells. This can activate HSC transformation into myofibroblasts and promote the development of liver fibrosis [11,12]. Some studies have shown that LSECs can induce the dedifferentiation type [13] or endothelial-to-mesenchymal transition (EMT) during the early stage of hepatic fibrosis [14]. Thus, how LSECs change during the model of liver fibrosis induced by schistosoma infection remains unknown.

In this study, a mouse model of *Schistosoma japonicum* infection was used. LSECs were isolated using gradient density centrifugation from the model mice. The changes in LSEC phenotype and function were detected by flow cytometry. The ultrastructure of LSECs was observed by scanning and transmission electron microscopes. The changes in EMT and fibrosis in LSECs and liver tissues were detected by qPCR and Western blot. This study sought to investigate the changes in LSECs in the infected model of schistosoma and the effect on liver fibrosis. These findings will be helpful to identify an effective strategy to treat liver fibrosis induced by *S. japonicum* infection.

## 2. Materials and Methods

### 2.1. Ethics Statement

Our experiments involving C57BL/6 mice were performed according to China’s Laboratory of Animal Welfare and Ethics Committee (LAWEC). The LAWEC Committee of the National Institute of Parasitic Diseases Chinese Center for Disease Control and Prevention approved the protocol (NIPD-2020-10).

### 2.2. Animals and Parasites

Female specific pathogen-free (SPF) C57BL/6 mice (6–8 weeks old; body weight 20 ± 2 g) were purchased from Shanghai Lingchang Biotechnology Co., Ltd. (Shanghai, China). Mice were housed in an SPF-grade animal room at our institute. The vector-borne tropical ward of our institute provided cerariae. Mice were percutaneously infected with cercariae by shaving the skin of the abdomen.

### 2.3. Reagents

Dulbecco’s phosphate-buffered saline (DPBS) and albumin from bovine serum (BSA) were purchased from Gibco (Grand Island, NY, USA). A Percoll cell separation solution was purchased from GE (Chicago, IL, USA), and collagenase from clostridium histolyticum was purchased from Sigma-Aldrich (St. Louis, MO, USA). Fluorescently conjugated antibodies, including PerCP-Cy5.5 rat anti-mouse CD45, FITC rat anti-mouse CD146, Fixable Viability Stain 575V, PE rat anti-mouse CD32b, Bv421 rat anti-mouse TGF-β and AF647 rat anti-mouse eNOS were purchased from Invitrogen Corporation (Waltham, MA, USA). Mouse CD146 kit Invitrogen™ was provided by Miltenyi Biotec Co., Ltd. (Bergisch Gladbach, Germany). Hyper ScriptTMIII RT SuperMix and Universal SYBR qPCR Mix were provided by Enzy Artisan (Shanghai, China).

### 2.4. Infection and Cell Isolation

A total of 60 C57BL/6 mice were randomly divided into an infected or uninfected group (30 mice/group). Mice in the infected group were anesthetized via an intraperitoneal injection of 1% sodium pentobarbital. Mice with shaved abdomen skin were percutaneously infected with 20 ± 1 cercariae. The uninfected group did not receive any treatment.

After anesthetizing the mice, 10 infected and 10 uninfected mice were sacrificed at 20, 28, and 42 days post infection, respectively. Mice were sterilized with 75% alcohol, secured to the board, and perfused with 1× Dulbecco’s phosphate-buffered saline (DPBS) to remove red blood cells. After perfusion, the liver was cut into fragments. Collagenase dissociated liver tissue into a single-cell suspension. Cells were separated by differential gradient centrifugation with 25%/50% Percoll solution [15]. The supernatant and lipid layer was discarded, and the cells were washed twice with DPBS. The red cells were lysed using BD Pharm Lyse™ lysing solution (Becton Dickinson and Company, Franklin Lakes, NJ, USA) to obtain hepatic non-parenchymal cells.

The concentrations of the hepatic non-parenchymal cells were adjusted to 1 × 10^7^ cells. We used an LSEC Isolation Kit (MiltenyiBiotec, Auburn, CA, USA) to isolate LSECs. The cellular suspension was centrifuged, and the supernatant was completely removed. The cell pellet was resuspended in 90 µL of buffer (phosphate-buffered saline, pH 7.2; 0.025% bovine serum albumin [BSA]; and 0.1 mM ethylenediaminetetraacetic acid [EDTA]). Next, 10 µL CD146 microbeads per 10^7^ total cells were added, and the mixture was incubated at 4 °C for 15 min. The cells were washed by adding 1 mL buffer and centrifuged at 300× *g* for 10 min, and the supernatant was completely removed using a pipette. The cell precipitates were resuspended with 500 μL buffer. The magnetic rack was placed, and the magnetic sorting column was placed in the magnetic field. Next, 500 μL buffer was added to wash the column, and the cell suspension was passed through the column. The column was washed with buffer three times. The sorting column was removed from the magnetic rack and placed on the appropriate collection tube. Then, 1 mL buffer was added to the column and the magnetic-labeled LSECs were flushed out through the plunger.

### 2.5. Flow Cytometry

The hepatic non-parenchymal cells were adjusted to 1 × 10^6^/mL using fluorescence-activated cell sorting (FACS) buffer (2% BSA and 2 mM EDTA in DPBS). The following antibodies were used in our experiments: Fixable Viability Stain 575V, CD45-Percp-cy5.5, CD146-FITC, CD32b-PE, and TGF-β Bv421. LSECs were defined as CD45^−^ CD146^+^.

Each 1 mL cell suspension was mixed with 1 μL Fixable Viability Stain 575V, incubated in the dark at room temperature for 15 min, and the cells were washed twice. Cells were stained with different combinations of antibodies for 30 min at room temperature (24–26 °C) in the dark, and washed with FACS buffer once. The fixation and permeabilization solution was added, incubated in the dark at 4 °C for 20 min, and the cells were washed twice. The cells were stained with intracellular anti-mouse TGF-β diluted antibody (antibody was diluted with 1 × Perm/Wash buffer) at 4 °C for 30 min, and washed twice. The cells were fixed in 1% paraformaldehyde at 4 °C for 20 min, and the cells were washed once. Next, 200 μL staining buffer was added to each sample, and the cells were suspended for detection. All experiments were carried out using a BD FACS Verse flow cytometer (BD Biosciences). Data were analyzed with FlowJo 10 software (TreeStar Inc., Ashland, OR, USA). The proportions of LSECs, CD32b^+^ LSECs, TGF-β^+^ LSECs, and eNOS expression were measured.

### 2.6. Electron Microscopy

Scanning electron microscope (SEM): after removing the red blood cells from the liver, an electron microscope fixation solution containing 2.5% glutaraldehyde was perfused into the mouse liver. After the liver had hardened, the edge of the right liver lobe was cut to approximately 2 mm × 2 mm and soaked into the electron microscope fixation solution for 2 h. The fixed samples were rinsed three times with phosphate buffer (0.1M) (PB, PH 7.4). The samples were fixed with 1% osmium acid at room temperature for 1–2 h avoiding light, and rinsed with PB three times. The samples were dehydrated, dried, and sprayed with gold for 30 s. The LSEC window pores were observed with SEM and photographed.

Transmission electron microscope (TEM): TEM samples were dehydrated twice with 30–100% alcohol and 100% acetone. The samples were embedded and polymerized at 60 °C for 48 h to prepare 60–80 μm ultra-thin slices. The samples were stained to avoid light with a 2% uranium acetate saturated alcohol solution for 8 min and to prevent carbon dioxide with a lead citrate solution for 8 min. After cleaning and drying, the samples were observed and photographed with a transmission electron microscope to evaluate the changes in the LSEC basement membrane.

### 2.7. Reverse Transcription Quantitative PCR

The total RNA from LSECs and mouse livers were extracted using TRIzol. Complementary DNA (cDNA) was synthesized using 1 μg total RNA with a Prime Script RT Master Mix (Takara, Shiga). RT-qPCR was used to determine the level of gene expression, including those in the liver samples (E-cadherin, N-cadherin, fibronectin, laminin, vimentin, α-SMA, collagen I, III, and IV) and LSEC samples (E-cadherin, VE-cadherin, Zonula occludens1, fibronectin, and α-SMA) using Fast SYBR Green master Mix (Bio-Rad, Hercules, CA, USA). The qPCR reaction system included 10 μL 2 × S6 Universal SYBR qPCR Mix, 1 μL upstream primers, 1 μL downstream primers, 3 μL cDNA, and 5 μL ddH_2_O. The primers used in this study are listed in Table 1, which were synthesized by Enzy Artisan Co., LTD (Shanghai, China). The qPCR reaction conditions were as follows: 95 °C for 30 s; 95 °C for 5 s, 60 °C for 30 s, over 38 cycles. After the circulating value (Ct) was obtained, the relative expression of the target gene was evaluated using 2^−ΔΔCt^.

### 2.8. Western Blot

The mouse liver tissues were collected and lysed using a radioimmunoprecipitation assay (RIPA) lysis buffer (Shanghai Epizyme Biomedical Technology Co., Ltd., Shanghai, China) supplemented with a protease inhibitor cocktail and EDTA (Beyotime Biotechnology, Shanghai, China). The lysates were centrifuged at 12,000× *g* for 10 min. The protein concentrations were detected using the BCA method. After boiling at 100 °C for 10 min, the samples were loaded into wells, and SDS-polyacrylamide gel electrophoresis was performed at 90 V for approximately 1 h. The protein strips were transferred to polyvinylidene fluoride (PVDF) membranes. After blocking, the membranes were incubated sequentially with primary and secondary antibodies. Anti-GAPDH (5174S, Cell Signaling Technology, CST, Danvers, MA, USA), anti-a-SMA (19245S, CST), and anti-Collagen I a1 (bs-7158R, Bioss) antibodies were used as the primary antibodies. A horseradish peroxidase-conjugated anti-mouse IgG antibody (7076S, CST) was used as the secondary antibody. Immunoreactive bands were visualized on digital images captured with a ChemiDoc MP Imaging System (Bio-Rad). The band intensities were quantified using Image J software (NIH, Bethesda, MD, USA).

### 2.9. Statistical Analysis

Data analysis was performed using GraphPad Prism Version 9.0.0 and SPSS 20.0 (IBM Corp., Armonk, NY, USA). Differences between the groups were assessed using a nonparametric one-way analysis of variance. Data were presented as the mean ± standard deviation. *p* < 0.05 indicated a significant difference.

## 3. Results

### 3.1. The Changes in the Proportion of LSECs in Mice Infected with S. japonicum

**Figure 1:**
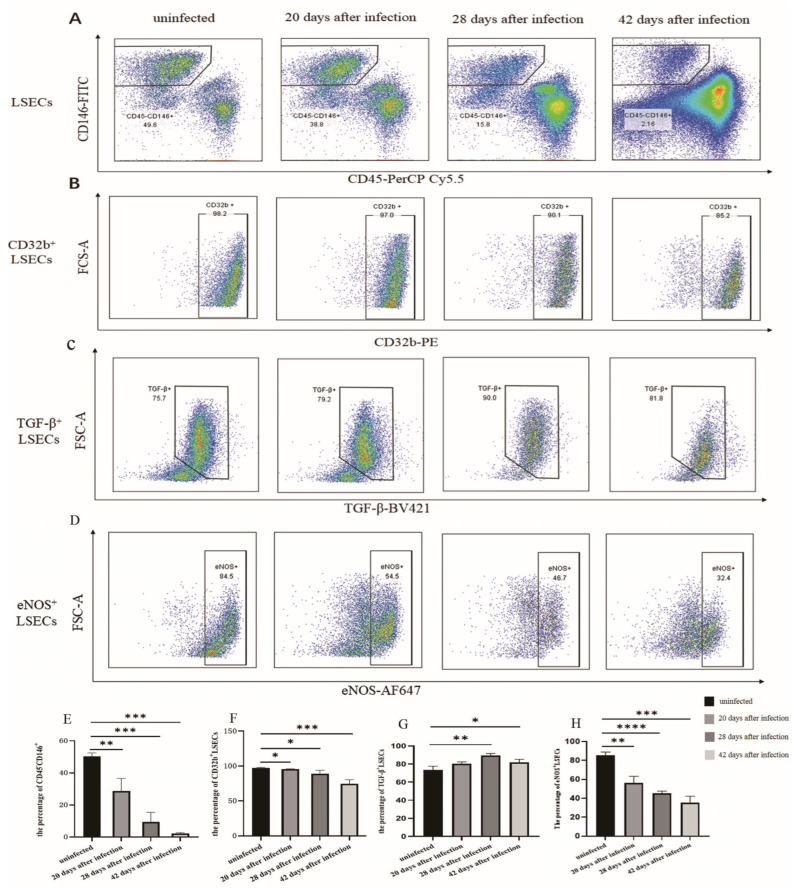
Changes in the LSEC phenotype after being infected with *S. japonicum*. (**A**) The population of LSECs in hepatic non-parenchymal cells after infection. (**B**) The population of CD32b^+^ LSECs in hepatic non-parenchymal cells after infection. (**C**) The population of TGF-β^+^ LSECs in hepatic non-parenchymal cells after infection. (**D**) The population of eNOS^+^ LSECs in the hepatic non-parenchymal cells after infection. (**E**) Histogram showing the proportion of LSECs (CD45^−^CD146^+^). (**F**) Histogram of the proportion of CD32b^+^ LSECs. (**G**) Histogram of the proportion of TGF-β^+^ LSECs. (**H**) Histogram of the proportion of eNOS^+^ LSECs. * *p* < 0.05; ** *p* < 0.01; *** *p* < 0.001; **** *p* < 0.0001.

The flow cytometry results showed that the percentage of LSECs (CD45-CD146^+^) in hepatic non-parenchymal cells was (28.70 ± 6.41)%, (9.43 ± 4.88)%, and (2.18 ± 0.49)% at 20, 28, and 42 days, respectively following *S. japonicum* infection. Compared with the uninfected group (50.40 ± 1.68)%, the proportion of LSECs decreased significantly (*p* < 0.01) (Figure 1A,D). After infection for 20, 28, and 42 days, the proportion of CD32b^+^ LSECs was (95.80 ± 0.28)%, (89.22 ± 4.03)%, and (74.82 ± 5.06)%, which was lower than that of the uninfected group (97.27 ± 0.58)% (Figure 1B,E). The TGF-β^+^ LSEC population was (80.20 ± 1.78)%, (88.37 ± 1.49)%, and (81.83 ± 3.55)%, which was higher than that of the uninfected group (73.37 ± 3.44)% (Figure 1C,F). The results indicated that the proportion of differentiated LSECs (CD32b^+^ LSECs) decreased, whereas the proportion of dedifferentiated LSECs (TGF-β^+^ LSECs) significantly increased after infection. The expression of eNOS in LSECs decreased significantly post infection during the 20th to 42th day post infection. This indicated that the secretion of NO in LSECs was decreased, and the function of LSECs was damaged.

### 3.2. Changes in the Number of Fenestrations of LSEC after Infection

**Figure 2:**
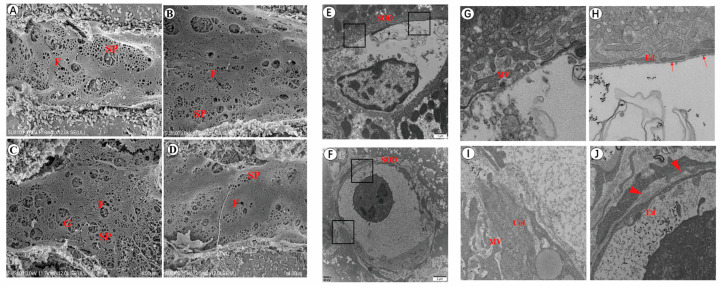
The structure of LSEC fenestration was altered in the liver of mice following infection. (**A**) The number of LSEC fenestration in the uninfected group. (**B**) The number of LSEC fenestration for 20 days after infection. (**C**) The number of LSEC fenestrations for 28 days post infection. (**D**) The number of LSEC fenestrations for 42 days post infection. (**E**) The structure of the LSEC basement membrane in the uninfected group. (**F**) The structure of the LSEC basement membrane for 42 days post infection. (**G**) The number of microvilli in the hepatic disc space of the uninfected mice (TEM magnification). (**H**) The structure of the LSEC basement membrane in the uninfected group (TEM magnification). (**I**) The number of microvilli and collagen in the hepatic disc space for 42 days post infection (TEM magnification). (**J**) The LSEC basement membrane structure for 42 days post infection (TEM magnification). SP, sieve plate; F, fenestration; G, gap; Ed, sinusoidal endothelial cells; SOD, space of Disse; MV, microvilli; Col, collagen; small arrow, fenestration; big arrow, basement membrane.

The SEM results showed that the LSECs of the uninfected mice displayed multiple fenestrations, which dispersed and connected to form a sieve plate. The number of fenestrations in LSEC decreased significantly post infection. In contrast, the number of LSECs without fenestration increased continuously (Figure 2A–D). The TEM results revealed that the number of microvilli in the disc space were reduced after infection for 42 days. A large amount of collagen was deposited in the hepatic disc space in the liver of infected mice. In the uninfected group, LSECs in the liver of the mice had obvious fenestration and did not have a basement membrane. After being infected for 42 days, fenestration disappeared, and the basement membrane appeared under the LSECs (Figure 2E–J).

### 3.3. The Changes of EMT in LSECs after Infection with S. japonicum

**Figure 3:**
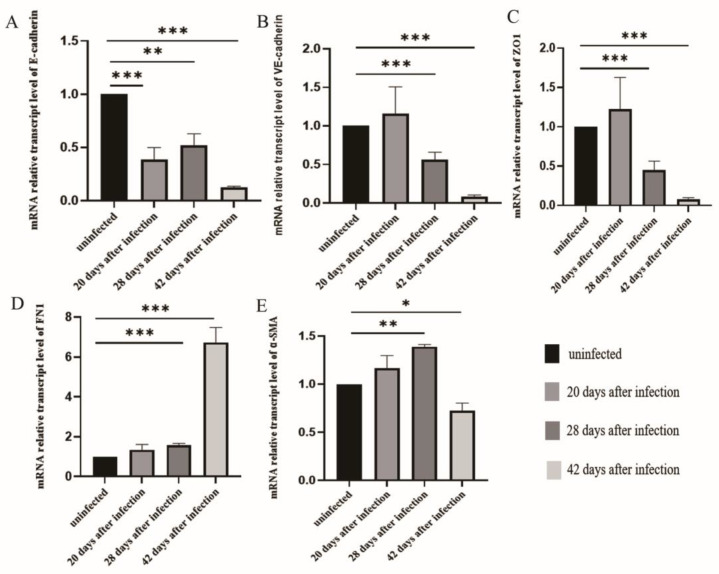
LSEC EMT after *S. japonicum* infection. (**A**) The level of E-cadherin mRNA expression in LSECs in mice post infection. (**B**) The level of VE-cadherin mRNA expression in LSECs in mice post infection. (**C**) ZO1 expression of LSECs in mice post infection. (**D**) FN1 expression in LSECs in mice post infection. (**E**) α-SMA expression in LSECs in the mice post infection. * *p* < 0.05; ** *p* < 0.01; *** *p* < 0.001.

The qPCR results showed that after infection for 20 days, the level of E-cadherin mRNA expression in the LSECs was significantly lower than that in the uninfected group (*p* < 0.05) (Figure 3A). However, the levels of VE-cadherin, ZO1, fibronectin, and α-SMA mRNA expression in LSECs did not differ from that of the uninfected group. Following infection for 28 and 42 days, the level of E-cadherin, VE-cadherin, and ZO1 mRNA expression in LSECs decreased (Figure 3A–C). In contrast, the levels of fibronectin and α-SMA mRNA expression increased significantly (Figure 3D–E). These results indicated that LSECs began EMT changes during the early stages after infection, and the LSECs transformed into fibroblasts during the middle and late stages, producing large amounts of α-SMA.

### 3.4. EMT and Liver Fibrosis in the Liver after Infection with S. japonicum

**Figure 4:**
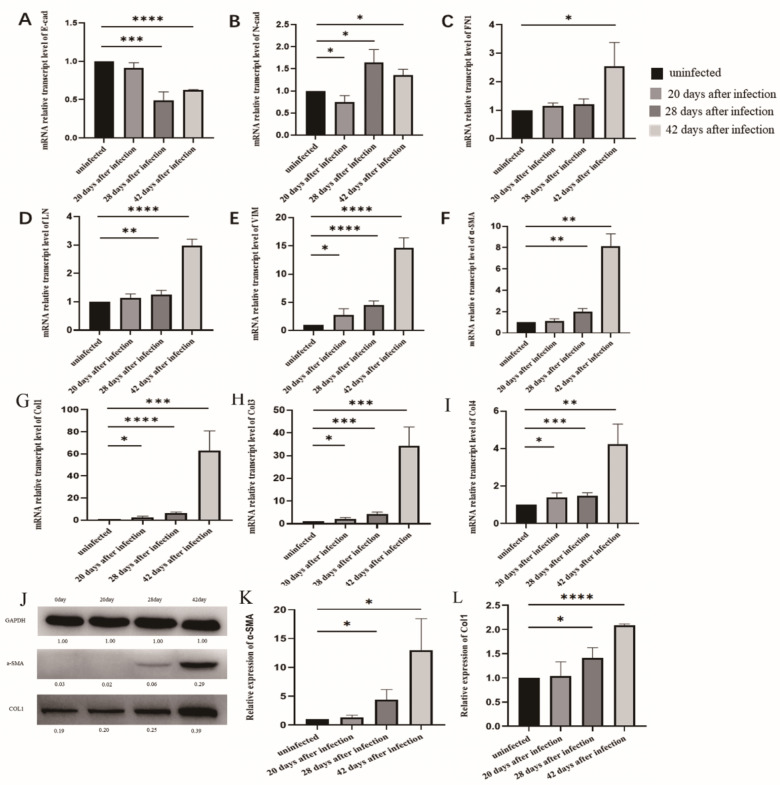
Fibrosis and EMT changes in the liver following infection with *S. japonicum*. (**A**–**E**) qPCR results of E-cadherin, N-cadherin, FN1, LN, and vimentin expression in the liver after infection. (**F**–**I**) qPCR results of α-SMA, collagen I, II, and III expression in the liver after infection. (**J**–**L**) Western blot results of α-SMA and collagen I expression in the liver after infection. * *p* < 0.05; ** *p* < 0.01; *** *p* < 0.001; **** *p* < 0.0001.

The qPCR results showed that the levels of mRNA markers associated with EMT (vimentin) and liver fibrosis (Collagen I, III, and IV) in the liver at 20 days post infection were significantly higher than those in the uninfected group (Figure 4E,G–I). After infection for 28 and 42 days, the level of E-cadherin mRNA decreased (Figure 4A), whereas the level of marker mRNA associated with EMT (N-cadherin, fibronectin, laminin, and vimentin) were increased significantly (Figure 4B–E). The levels of marker mRNA associated with liver fibrosis, including α-SMA, collagen I, III, and IV, were increased at 28 and 42 days post infection (Figure 4F–I). The Western blot results showed that the protein levels of α-SMA and collagen I were increased consistently at 28 and 42 days post infection (Figure 4J–L). These results indicated that at 20 days post infection, the liver began to undergo moderate changes in fibrosis and EMT. After infection for 28 and 42 days, the changes in fibrosis and EMT in the liver became more significant.

## 4. Discussion

Schistosomiasis is a globally distributed neglected tropical disease, which primarily occurs in tropical and subtropical regions [16]. Worldwide, approximately 236 million people are infected with schistosomes, 90% of which are located in sub-Saharan Africa, and results in approximately 300,000 deaths per year [17,18]. In China, according to the national schistosomiasis epidemic bulletin in 2020, although schistosomiasis has a low prevalence, there remains a large number of patients with schistosomiasis liver fibrosis [2]. At present, there is no effective treatment for liver fibrosis induced by schistosoma infection. Therefore, elucidating the regulatory mechanism of schistosomiasis liver fibrosis is essential to providing potential targets for the treatment of liver fibrosis.

After being infected with *S. japonicum*, the eggs are deposited in the host liver. Several antigens from the schistosomula and soluble eggs that are released induce both immune and inflammatory responses [19,20]. Some cells (e.g., hepatocytes, hepatic stellate cells, hepatic sinusoidal endothelial cells, and bile duct epithelial cells) transform into myofibroblasts (MFB), releasing large amounts of extracellular matrix (ECM) [21]. The mass transformation to MFB represents a key link in the development of hepatic fibrosis. A large amount of ECM deposits in the liver tissue leads to the formation of schistosomiasis liver fibrosis and seriously affects human health [22].

LSECs are at the highest proportion in mouse livers, accounting for approximately 70% of liver non-parenchymal cells [23]. Moreover, LSECs play an essential role in maintaining the balance between liver regeneration and fibrosis [10]. LSECs have differentiation and dedifferentiation phenotypes. The differentiated LSECs have a normal function, which are rich in fenestration and lack a complete basement membrane. The permeability of hepatic sinuses depends on this unique structure, which promotes the exchange of nutrients and gases between cells. Nitric oxide synthase (eNOS) activity is high in differentiated LSECs, which produce and release NO, and maintain the resting state of HSCs [8]. In chronic liver injury, the fenestration diameter of LSECs decreased, and a complete basement membrane gradually formed. This results in changes to LSEC dedifferentiation [12]. During the early stage of non-alcoholic liver injury, chronic hepatitis, and other models, LSECs exhibit changes in dedifferentiation [13,24].

The key feature of MFB activation is epithelial–mesenchymal transition (EMT) [21,25,26,27]. During the change in EMT, epithelium acquires mesenchymal properties, which plays an important role in tissue repair, inflammation, fibrosis, and other processes [28,29]. EMT is an important source of myofibroblasts [10]. Growing evidence shows that when EMT is dominant in tissues, liver tissues progress toward fibrosis [27]. Dedifferentiated LSECs transform into myofibroblast-like cells through EMT and secrete a large amount of fibronectin and α-SMA to promote fibrosis formation [30,31,32]. Therefore, maintaining LSECs with a differentiated phenotype is an effective strategy for reversing hepatic fibrosis.

After infection, LSECs developed a dedifferentiated phenotype and changed into mesenchymal cells. CD32b is an essential indicator of differentiated LSEC, and high levels of TGF-β were detected in dedifferentiated LSEC [12,33]. Following infection for 20, 28, and 42 days, the proportion of total LSECs (CD45^−^CD146^+^) and differentiation phenotype CD32b^+^ LSECs in infected mice decreased continuously, whereas the dedifferentiation phenotype (TGF-β^+^ LSECs) increased significantly. Following infection, the level of eNOS protein expression in LSECs decreased significantly, indicating that the amount of NO secreted by LSECs had decreased significantly. This finding suggested that the ability of LSECs to maintain the resting state of HSCs was reduced. Moreover, the number of fenestrations in LSECs decreased and the basement membrane formed. This finding suggested that LSECs converted to a dedifferentiated phenotype in the middle and late stages of infection. EMT is a dynamic pathological process. Some of the dedifferentiated LSECs underwent further EMT changes and transformed into mesenchymal cells, producing a large amount of ECM. The level of epithelial marker mRNA, including E-cadherin, VE-cadherin, and Zo1, decreased, while mesenchymal markers (FN1 and α-SMA) increased during the early stages. Additionally, they changed more significantly during the middle and late stages of infection. These results indicated that the dedifferentiation and EMT in LSECs began during the early stages of infection (20 days post infection). With the development of infection, the extent of pathological changes aggravated in the LSECs.

Pathological changes in LSECs could further promote the progression of liver fibrosis induced by *S. japonicum* infection. On the one hand, dedifferentiated LSECs could induce HSC activation. Dedifferentiated LSECs reduced NO secretion but greatly increased TGF-β secretion post infection. NO benefits the maintenance of HSC quiescence [11,34], whereas TGF-β is a classical cytokine that activates HSCs [35]. Activated HSCs were the primary source of MFB [36,37]. Therefore, dedifferentiated LSECs had transformed into a pro-inflammatory phenotype, which could indirectly promote liver fibrosis by activating HSCs. On the other hand, dedifferentiated LSECs could directly undergo EMT changes, transform into MFB, produce a large number of ECM, and promote hepatic fibrosis [38,39]. Maintaining the differentiated phenotype of LSEC is an effective treatment for hepatic fibrosis. Some studies have shown that the administration of statin treatment or actin depolymerization to maintain the LSEC phenotype could decrease portal pressure and improve NASH features in an early NASH model [33,40]. Long noncoding RNAs can interact with EZH2, and maintain LSEC differentiation through KLF2-eNOS-sGC pathway and alleviate CCl_4_-induced liver fibrosis [41].

It has been well established that egg deposition in the liver releases soluble egg antigens and recruits lymphocytes, leading to the formation of granuloma and liver fibrosis [42,43]. However, our experiments indicated that egg deposition in the liver was not the only cause of liver fibrosis. LSEC injury during the early stage of infection also promoted the progression of liver fibrosis. It was established that *S. japonicum* lay eggs about 24 days post infection. The pathological changes in LSECs occurred earlier than schistosome oviposition. Pathological lesions appeared in LSECs on the 20th day or earlier after infection. Pathological injury to LSECs could also induce EMT and fibrosis changes in the liver tissue. The qPCR and Western blot results showed that markers of EMT and fibrosis in the liver changed during the early stage (20th day post infection) and aggravated in the middle and late stages of infection. The pathological changes in the liver were consistent with that of LSECs. Thus, histological changes in LSECs induced by *S. japonicum* infection are also important factors for promoting liver fibrosis.

However, our study only simultaneously observed EMT and fibrosis changes in LSEC and liver tissue in schistosomiasis and did not determine whether EMT changes occurred earlier than liver fibrosis. It also remains unknown whether EMT changes cause liver fibrosis. Therefore, further experiments are needed to explore the role of EMT in liver fibrosis.

## 5. Conclusions

In the present study, we identified changes in the pathology and functional impairment of LSEC following an infection with *S. japonicum*. These changes predated schistosome oviposition. The changes in LSEC were consistent with the process of liver fibrosis induced by *S. japonicum* infection. This indicates that LSECs are involved in the regulation of schistosomiasis liver fibrosis. Therefore, using drugs or Lnc RNA to target the pathological changes in LSECs represents a promising strategy that can alleviate or reverse schistosomiasis liver fibrosis.

## Figures and Tables

**Table 1:** Primer sequences for qPCR.

Gene Name	Primer Sequence (5′→3′)
GAPDH	F:CATCACTGCCACCCAGAAGACTG
	R:ATGCCATGAGCTTCCCGTTCAG
E-cadherin	F:GGTCATCAGTGTGCTCACCTCT
	R:GCTGTTGTGCTCAAGCCTTCAC
VE-cadherin	F:GAACGAGGACAGCAACTTCACC
	R:GTTAGCGTGCTGGTTCCAGTCA
Zonula occludens1 (ZO1)	F:GTTGGTACGGTGCCCTGAAAGA
	R:GCTGACAGGTAGGACAGACGAT
Fibronectin (FN)	F:GGTCCTCTCCTTCCATCTCCTTAC
	R:GGACCCCTGAGCATCTTGAGTG

## Data Availability

Data are contained within the article.
